# Passive recharge burst spinal cord stimulation for the treatment of refractory nonsurgical low back pain: 24-month results from a prospective randomized controlled trial and predictors of success

**DOI:** 10.1016/j.xnsj.2026.100911

**Published:** 2026-06-08

**Authors:** James J Yue, Steven Falowski, Edward Tavel, Robert Heros, Anne Christopher, Denis Patterson, Robert Funk, Ibrahim Mohab, Sayed Wahezi, Jacqueline Weisbein, Christopher J Gilligan, Ajay Antony, Michael Fishell, Jessica Jameson, Chi Lim, Nathan Miller, Derron Wilson, Patrick Buchanan, David Dickerson, Keith Scarfo, Robert Levy, Mehul J Desai, Edward Braun, Susan Moeschler, Julie Pilitsis, Scott Kreiner, Jijun Xu, Jonathan Duncan, Kenneth Candido, Michael J Dorsi, Rafe Sales, Todd Lansford, Martin E Weinand, Nrupen Baxi, Jason Garber, James Forage, Albert E Telfeian, Haddad Souheil, Bram Blomme, Natalie Brill, Gayle Johnson, Timothy Deer

**Affiliations:** a Connecticut Orthopaedic Specialists, Hamden, CT, United States; b Neurosurgical Associates of Lancaster, Lancaster, PA, United States; c Clinical Trials of South Carolina, Charleston, SC, United States; d Spinal Diagnostics, Tualatin, OR, United States; e Saint Louis Pain Consultants, Creve Coeur, MO, United States; f Nevada Advanced Pain Specialists, Reno, NV, United States; g Indiana Spine Group, Indianapolis, IN, United States; h Department of Anesthesiology and Pharmacology, The University of Arizona, Tucson, AZ, United States; i Montefiore Medical Center, New York, NY, United States; j Napa Valley Orthopaedic Medical Group, Napa, CA, United States; k Brigham & Women’s Hospital, Boston, MA, United States; l The Orthopaedic Institute, Gainesville, FL, United States; m Advanced Pain Care, Henderson, NV, United States; n Axis Spine Center, Coeur d’Alene, ID, United States; o Carolina Orthopaedic & Neurosurgical Associates, Spartanburg, SC, United States; p Coastal Pain & Spinal Diagnostics Medical Group, Carlsbad, CA, United States; q Goodman Campbell Brain & Spine, Greenwood, IN, United States; r Spanish Hills Interventional Pain Specialists, Camarillo, CA, United States; s Endeavor Health, Evanston, IL, United States; t Brown University Health, Providence, RI, United States; u Anesthesia Pain Care Consultants, Tamarac, FL, United States; v International Spine, Pain & Performance Center, Washington, DC, United States; w Kansas University Medical Center, KS, United States; x Mayo Clinic, Rochester, MN, United States; y Florida Atlantic University, Boca Raton, FL, United States; z Barrow Brain & Spine, Phoenix, AZ, United States; aa The Cleveland Clinic Foundation, OH, United States; bb Burkhart Research Institute for Orthopaedics, San Antonio, TX, United States; cc Chicago Anesthesia Associates, SC, Chicago, IL, United States; dd Neurosurgery, University of California Los Angeles, Los Angeles, CA, United States; ee Summit Spine Institute, Portland, OR, United States; ff South Carolina Sports Medicine and Orthopaedic Center, Charleston, SC, United States; gg Montefiore Medical Center, Albert Einstein College of Medicine, Bronx, NY, United States; hh Center for Spine & Brain Surgery, Las Vegas Neurosurgical Institute, Las Vegas, NV, United States; ii The Spine and Brain Institute, Henderson, NV, United States; jj Department of Neurosurgery, Warren Alpert Medical School, Brown University, Providence, RI, United States; kk Abbott Neuromodulation, Austin, TX, United States; ll The Spine and Nerve Center of the Virginias, Charleston, WV, United States

**Keywords:** Nonsurgical low back pain, Spinal cord stimulation, Passive recharge burst stimulation, Long-term outcomes, Predictor analysis, Neuropathy, Psychological distress, Paddle leads, Percutaneous leads

## Abstract

**Background:**

Spinal cord stimulation (SCS) is an established treatment for chronic pain, but long-term results are limited. The objective of this manuscript was to evaluate the 24-month durability of outcomes for patients with nonsurgical refractory chronic low back pain (NS-CLBP) treated with implanted burst SCS, including common diagnoses of NS-CLBP, and predictors of SCS success.

**Methods:**

The DISTINCT study was a prospective, multicenter, randomized, controlled trial that investigated the use of SCS for NS-CLBP compared to conventional medical treatment alone. Efficacy and safety data were collected after the optional crossover at 6 months. Univariable and multivariable regression analyses were performed for predictor analyses at 24 months. Dependent variables were pain intensity (NRS), back pain-related disability (ODI), and pain catastrophizing (PCS). Independent variables were selected baseline characteristics, including the PainDetect questionnaire to assess pain as neuropathic (vs. mechanical/nociceptive).

**Results:**

NRS responder rates (≥50% reduction) at 24 months for SCS and crossover patients were of 77% and 79%, respectively. Secondary outcomes indicated significant sustained improvements in ODI, PCS, and reduced healthcare utilization. Six serious adverse events were reported and resolved without sequelae. Improvements on the NRS, ODI, and PCS for common subetiologies mirrored those of the overall NS-CLBP group. After adjusting for covariates, higher baseline PCS scores and leg pain were predictors of super-responders, or patients who responded to all 3 scales. Patients with predominantly neuropathic pain reported more robust 24-month ODI and PCS outcomes than patients with mechanical/nociceptive pain. Patients reported similar NRS improvements regardless of pain type.

**Conclusions:**

Implanted passive recharge burst SCS provides a significant and sustained therapeutic effect 2 years after implantation in patients with NS-CLBP. Even patients with the highest symptom burden report success at 24 months. Although neuropathic pain increases the magnitude of burst SCS success in NS-CLBP, particularly regarding disability and catastrophizing, patients with nociceptive pain also obtain substantial benefit.

## Highlights

• Implanted passive recharge burst spinal cord stimulation provides a significant and sustained therapeutic effect 2 years after implantation in patients with nonsurgical chronic low back pain (NS-CLBP).
• Improvements in patient-reported outcomes for common diagnoses of NS-CLBP are consistent with those of the overall NS-CLBP group.
• Higher baseline pain catastrophizing scores and the presence of leg pain were predictive of super-responders at 24 months, or patients who responded on pain, catastrophizing, and disability due to low back pain.
• Neuropathic pain increases the magnitude of burst SCS success in NS-CLBP, although patients with nociceptive/mixed pain also obtain substantial benefit.

## Background

Chronic low back pain (CLBP) is the leading cause of disability worldwide, affecting over half a billion people [1]. A significant subgroup of patients with refractory CLBP have conditions which are not treatable with corrective surgery. Nonsurgical CLBP (NS-CLBP) is not a single condition but is often multifactorial. Most NS-CLBP diagnoses results from age-related degenerative changes of various anatomical structures of the spine including anterior column and regional and/or global changes in spinal alignment, such as scoliosis or spondylolisthesis. Most patients suffer from multiple, sometimes overlapping, sources of pain [2].

Given the concerns about the invasiveness, cost, and effectiveness of spinal surgery, and the challenges of treating patients with chronic low back pain in the absence of a clearly identified surgical target, there is a need for interventions that offer pain relief and long-term functional recovery, including spinal cord stimulation (SCS) [3]. SCS has been used to treat chronic pain for almost 60 years, and the mechanisms of action have been described in several publications [[4], [5], [6]]. To date, studies show that SCS is effective in the treatment of CLBP due to postsurgical pain and is superior to conservative medical management and repeat surgery [[7], [8], [9], [10]]. Passive recharge burst stimulation is an advancement in SCS over traditional tonic stimulation and has increased its overall effectiveness (vs. tonic SCS) as a treatment modality for chronic pain, because it is found to alter both sensory and emotional pathways in the brain [[11], [12], [13], [14]].

The DISTINCT randomized controlled clinical trial (RCT) evaluated passive recharge burst SCS for back pain patients who have not undergone and are not candidates for lumbar spine surgery [15]. In this report, we present 24-month results of the DISTINCT study. Additionally, we performed a multivariable regression analysis across the different subtypes of NS-CLBP including patient characteristics to better understand optimal patient selection for passive recharge burst SCS treatment.

## Methods

### Study design

The DISTINCT study was a prospective, multicenter RCT with an optional crossover component after the 6-month primary endpoint to evaluate the efficacy of burst SCS in combination with conventional medical management (CMM) in the treatment of chronic axial low back pain compared to CMM alone. The study was conducted at 30 sites throughout the United States (US). The study is registered on ClinicalTrials.gov; NCT04479787. An institutional review board approved study documents and all updated versions. This study was conducted in accordance with US FDA regulatory requirements, Good Clinical Practice, and the ethical principles of the Declaration of Helsinki. The sponsor had final approval on study design, which was developed in collaboration with investigators.

### Participants

All patients signed informed consent before enrollment. Full inclusion and exclusion criteria have been presented previously [15]. Advanced imaging (magnetic resonance imaging and/or computed tomography) of the spine obtained within 12 months was reviewed by an independent orthopedic spine surgeon before enrollment and randomization to confirm lack of an identifiable pathology that could effectively be treated with surgery. Key eligibility criteria included a primary complaint of back pain, with or without leg pain, and with moderate-to-severe back pain-related disability. All patients had undergone a variety of unsuccessful conservative care treatments and were considered refractory to conservative care.

The term persistent spinal pain syndrome type 1 (PSPS-1) was introduced in 2021 to designate patients with chronic axial pain and/or radicular symptoms of spinal origin in individuals who have not undergone spinal surgery [16]. Since the patients in this study were not eligible for spinal surgery, it concerns a specific subpopulation of PSPS-1. Patients were referred to in this document as nonsurgical chronic low back pain.

At the start of the study, investigators were asked to indicate all pain diagnoses on a case report form. Options included chronic nonspecific low back pain, discogenic pain, degenerative disc disease, lumbar disc herniation, lumbar facet arthropathy, lumbar radiculopathy, lumbar spinal stenosis, lumbar spondylosis, mechanical low back pain, spondylolisthesis, and scoliosis. Investigators could specify any other pain diagnoses not included in the options above.

### Randomization, crossover, and concealment

Subjects were randomized in a 3:2 ratio to either the burst SCS arm or the CMM arm, stratified by site. The primary endpoint was evaluated at 6 months after which patients had the option to cross over to the other treatment arm. Patients crossing over from CMM to SCS had to receive their permanent implant before the 12-month follow-up visit and continued with the follow-up schedule. All assignments were allocated using an electronic data capture system. The subjects, site personnel, and some sponsor personnel were aware of the treatment assignment. Statisticians were blinded to treatment assignment until performing the primary end-point analysis of the randomized cohort (presented in [15]).

### Procedures

BurstDR-capable implantable pulse generators (IPGs), along with relevant leads and accessories, were used in accordance with FDA-approved instructions for use. All subjects randomized to the burst SCS arm underwent a 4–7 days trial period, with lead location per the physician’s customary practice for treating low back pain and underwent implant procedures only if they experienced at least 50% pain relief. Patients crossing over from CMM to the SCS arm followed the same standard trial procedure. Burst SCS stimulation parameters were configured using the clinician programmer. Per current standard of care, burst stimulation was delivered at low amplitudes and intermittently in a 1:3, 1:6, or 1:12 ratio (30 second on and 90, 180, or 360 second off). Percutaneous or paddle leads were used at the implant depending on the surgeon’s preference. A choice of recharge-free or rechargeable IPG was used based on surgeon and patient preference (Proclaim or Prodigy).

CMM included medication optimization, supervised noninterventional therapy, and nonoperative interventional treatments. Specifically, at each follow-up visit, the type of ancillary care the patient had received since the last study visit was documented on a case report form. Patients could choose from “None,” “Physical therapy,” “Chiropractic therapy,” “Massage therapy,” “Acupuncture,” “Injection therapy,” “Radiofrequency ablation,” and “Other” with the option to specify.

Patients were followed in-clinic for required study visits at 1, 3, 6, 9, 12, 18, and 24 months and via phone call or optional clinic visit at 15 and 21-months.

### Outcomes

The study endpoints incorporated outcome measures and associated clinically meaningful improvements per the IMMPACT guidelines [17]. Responders were defined by the primary endpoint of at least a 50% reduction in low back pain as measured by the numerical rating scale (NRS). Other endpoints assessed included back pain-related physical disability (Oswestry Disability Index [ODI]), pain-related emotional distress (Pain Catastrophizing Scale [PCS]), quality of life (Patient-reported Outcome Measure Information System [PROMIS-29] domains, Patient Global Impression of Change (PGIC), satisfaction with therapy, pain-related medication usage, and healthcare utilization. All adverse events were reported by the investigators throughout the study and reviewed and adjudicated by an independent clinical events committee.

Abbott designed and owned the data capture system, but data collection and data entry were performed independently by the investigating sites.

### Statistical analysis

The sample size calculation, primary analysis at 6 months, and additional analysis at 12 months following permanent implant have been described previously [15,18]. All endpoints are summarized through 24 months. Continuous variables are presented as means ± standard deviations. Categorial variables are summarized as percentages. Clinically meaningful changes were defined as at least a 50% reduction in pain on the NRS and at least a 13-point reduction, at least 1 category improvement, or score ≤20% on ODI in functional disability [19]. A PCS responder was defined as a patient who was either clinically catastrophizing at baseline (PCS score ≥30) and reported a score of <30 at 24 months or reported a 40% decrease in score at 24 months [20,21]. Missing data was handled through complete case analysis. The study protocol and statistical analysis plan were prespecified. Statistical analyses were conducted according to this predefined plan.

In addition to the analyses stipulated in the protocol, this manuscript also presents post-hoc exploratory analyses. Univariable and multivariable regression were performed using generalized linear models for absolute change in NRS, ODI, and PCS and logistic regression for super-responder status (defined as patients responding on all 3 scales). Stepwise regression was used to select the best model, with all effects with p-values ≤ .1 in the univariable analysis being evaluated in a multivariable analysis.

For the multivariable regression models, both forward and backward stepwise methods were tested. Stepwise regression modelling was chosen to facilitate interpretation, given the exploratory nature of these analyses. Across all models, 22 baseline variables were initially assessed as candidate predictors. For the stepwise logistic regression model evaluating super-responder status, only 5 baseline variables were included in the multivariable model. Given that 55 patients were classified as nonresponders, this approach maintained an event-to-variable ratio exceeding the commonly recommended threshold of 10 [22].

All evaluated predictors with 95% confidence intervals and p-values are shown in Supplementary Material Table C1 and include various aspects of NS-CLBP diagnosis and baseline clinical profile. Statistically significant effects of the univariable and multivariable analyses are presented in Table 1, Table 2, respectively. One possible predictor was the PainDetect questionnaire, a validated screening tool for differentiating neuropathic (from nociceptive/mechanical) pain. Both continuous and categorical values were considered. Scores range from 0–38, with total scores ≤12 indicating nociceptive/mechanical pain, 13–18 possible neuropathic pain (or mixed pain), and ≥19 representing >90% likelihood of neuropathic pain. For ease of interpretation, effects are presented as factors associated with greater absolute change or a higher responder rate for most outcomes. For example, if presence of a subetiology decreased the likelihood of success, this was presented as “absence of etiology.”

**Table 1:** Predictors of response at 24 months (effects with p-values ≤.1 for at least 1 variable); univariable analysis.

Clinical characteristics*	NRS change		ODI change		PCS change		Super-responder	
	Estimate [95% CI]	p-value	Estimate [95% CI]	p-value	Estimate [95% CI]	p-value	Odds ratio [95% CI]	p-value
Baseline clinical outcomes								
Baseline NRS score	1.059 [0.716, 1.402]	<.0001	3.918 [1.111, 6.725]	.006	0.653 [−1.670, 2.976]	.582	1.199 [0.864, 1.664]	.277
Baseline ODI score	0.026 [−0.005, 0.056]	.096	0.742 [0.555, 0.930]	<.0001	0.135 [−0.047, 0.317]	.146	1.019 [0.993, 1.046]	.155
Baseline PCS score	0.042 [0.009, 0.074]	.012	0.366 [0.124, 0.608]	.003	0.938 [0.814, 1.062]	<.0001	1.037 [1.007, 1.068]	.0162
Pain diagnosis								
Lumbar radiculopathy	0.090 [−0.765, 0.944]	.837	3.827 [−2.514, 10.168]	.238	−4.965 [−10.047, 0.118]	.057	1.080 [0.526, 2.214]	.834
Absence of lumbar spinal stenosis	0.823 [−0.135, 1.781]	.094	5.567 [−1.636, 12.769]	.132	0.454 [−5.353, 6.260]	.878	1.668 [0.742, 3.754]	.216
Decreasing number of pain diagnoses	0.252 [−0.028, 0.532]	.078	0.409 [−1.664, 2.482]	.699	−0.059 [−1.749, 1.632]	.946	1.122 [0.888, 1.418]	.335
Had ≤ 3 pain diagnoses	1.165 [0.206, 2.124]	.018	4.062 [−3.170, 11.294]	.272	−1.997 [−7.857, 3.863]	.504	1.406 [0.626, 3.161]	.410
Presence of leg pain (unilateral/bilateral)	0.480 [−0.419, 1.380]	.296	7.914 [1.247, 14.580]	.021	3.501 [−1.925, 8.928]	.207	2.500 [1.156, 5.407]	.020
PainDetect questionnaire (reference for categorical scores: nociceptive/mechanical pain [≤ 12])								
Continuous scores	0.0524 [−0.005, 0.110]	0.074	0.853 [0.452, 1.254]	<0.0001	0.548 [0.213, 0.884]	.001	1.069 [1.015, 1.126]	.011
Categorical scores Mixed pain (PDQ 13–18)	0.504 [−0.456, 1.464]	0.345	3.499 [−3.366, 10.364]	0.0005	1.774 [−3.896, 7.445]	.024	0.978 [0.437, 2.189]	.048
Categorical scores Neuropathic pain (PDQ ≥ 19)	0.711 [−0.308, 1.730]		14.556 [7.360, 21.753]		8.246 [2.279, 14.214]		3.026 [1.170, 7.825]	

NRS, numerical rating scale; ODI, Oswestry Disability Index; PCS, pain catastrophizing scale; PDQ, PainDetect Questionnaire.

⁎ For clarity, effects are presented as factors that increased the likelihood of greater absolute change or a higher responder rate for most outcomes. For example, if a subetiology decreased the likelihood of success, it is presented as “absence of etiology.”

**Table 2:** Significant predictors of response at 24 months; multivariable analysis.

Clinical characteristics	NRS change		ODI change		PCS change		Super-responder	
	Estimate [95% CI]	Multivariable	Estimate [95% CI]	Multivariable	Estimate [95% CI]	Multivariable	Odds ratio [95% CI]	Multivariable
Baseline clinical outcomes								
Baseline NRS score	1.059 [0.716, 1.402]	<0.0001	NA	NS	NA	NS	NA	NS
Baseline ODI score	NA	NS	0.742 [0.555, 0.930]	<0.0001	NA	NS	NA	NS
Baseline PCS score	NA	NS	NA	NS	0.938 [0.814, 1.062]	<0.0001	1.040 [1.009, 1.072]	0.0104
Pain diagnosis								
Presence of leg pain (unilateral/bilateral)	NA	NS	NA	NS	NA	NS	2.763 [1.241, 6.153]	0.013

NA, not applicable; NRS, numerical rating scale; NS; nonsignificant; ODI, Oswestry Disability Index; PCS, pain catastrophizing scale.

The sponsor statistician performed all data analysis unblinded after the primary endpoint analysis. Sponsor involvement did not influence data interpretation or the reporting of results. All data analyses were performed using SAS version 9.4 (SAS Institute Inc). The figures were created in GraphPad Prism (Dotmatics).

## Results

### 24-month study results

#### Summary of participation and crossover

The study enrolled 270 patients between September 14, 2020 and September 16, 2021. In total, 269 subjects were randomized: 162 to SCS and 107 to CMM. Of the patients randomized to SCS, 142 were trialed and 115 (81%) patients received a permanent implant. Baseline demographics, diagnoses and other characteristics were well-balanced between the groups and have been published previously [15]. After the 6-month endpoint, no patients randomized to SCS elected to cross over to CMM, while 86% (70/81) of patients crossed over from the CMM cohort to the SCS cohort (the crossover rate was 65% (70/107) when all patients randomized to CMM were considered). Of these, 66 were trialed and 58 (88%) received a permanent implant. At the end of the study, 98 patients in the SCS group and 48 patients in the CMM crossover group, respectively, had completed their 24- and 18-month follow-up after permanent implant. The final visit was completed on February 26, 2024.

**Fig. 1:**
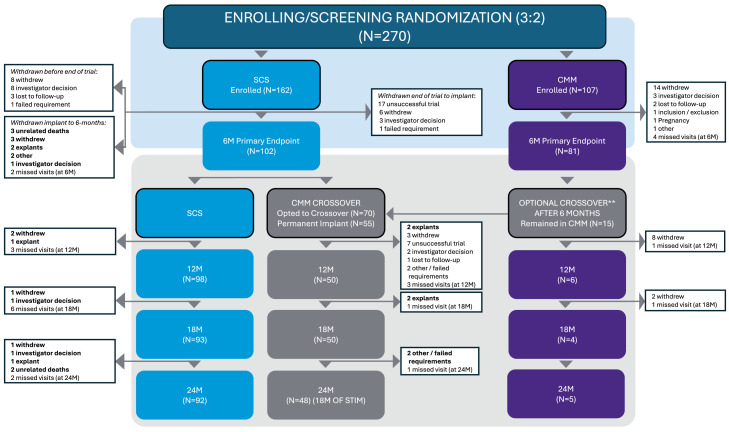
CONSORT diagram subject disposition from enrollment through 24 months. Reasons for withdrawal of patients with an SCS implant are shown in bold. CMM, conventional medical management; SCS, spinal cord stimulation.

Reasons for withdrawal are presented in Fig. 1. Although 269 patients were initially randomized in the DISTINCT study, 170 ultimately underwent permanent SCS implantation (including patients originally randomized to SCS and those who crossed over). Among the 170 implanted patients, 27 exited the study prior to their final scheduled follow-up, corresponding to an exit rate of 16%. Of the 27 patients who exited the study prematurely, the most common reasons were explantation of the SCS system (n = 8), all related to an adverse event or insufficient pain relief, subject withdrawal (n = 5); 3 patients no longer wished to participate and 2 patients felt the device was not providing benefit, and patient death (n = 5), all unrelated to the device or the procedure.

#### Pain intensity reduction

**Fig. 2:**
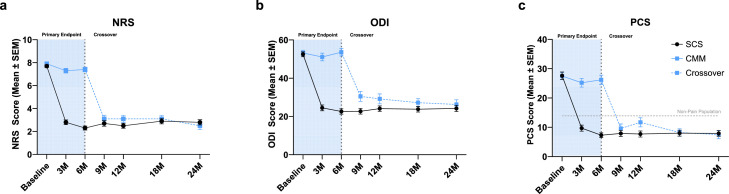
Change in NRS, ODI, and PCS from baseline through 24 months. CMM; conventional medical management; NRS, numerical rating scale; ODI, Oswestry Disability Index; PCS, pain catastrophizing scale; SCS, spinal cord stimulation; SEM, standard error of mean.

Outcomes through 12 months, including the primary endpoint analysis at 6 months, have been reported previously. At 24 months, 77% (70/91) of SCS patients and 79% (38/48) of crossover patients reported a ≥ 50% pain reduction. Approximately a third of the patients reported a substantial decrease in pain (≥80%) in both SCS (26/91) and crossover (17/48) groups. The score in the SCS group decreased from 7.7 ± 1.2 at baseline to 2.8 ± 2.2 at 24 months. This represents a 4.9 ± 2.5 decrease at 24 months. The CMM crossover arm reported a 24-month NRS score of 2.5 ± 2.3, which represents an average pain decrease of 5.3 ± 2.4 at 24 months (Fig. 2, Left). Eleven patients chose not to switch to the SCS arm of which 5 completed 24-month follow-up. Given this low number, efficacy results from this CMM group after 6 months are not presented because no meaningful comparisons with the SCS/crossover groups can be made.

#### Other patient-reported outcome measures

Similarly, secondary outcomes have previously been reported for ODI, PCS, and health-related quality of life (PROMIS-29) at 6 and 12 months.

At 24 months, 72.4% (63/87) and 63.2% (55/87) of patients in the SCS arm reported a decrease of ≥13 and ≥20 points, respectively, in their ODI score. The ODI score for the SCS arm decreased by 27.8 ± 17.7 points. After crossover, 73.3% (33/45) and 68.9% (31/45) of patients reported a decrease of ≥13 and ≥20 points, respectively, in their ODI score at 24 months. The ODI score for the CMM crossover arm decreased by 28.2 ± 19.1 points (Fig. 2, Middle).

The PCS score decreased from 27.6 ± 13.6 at baseline to 7.9 ± 9.0 at 24 months and from 27.5 ± 12.5 at baseline to 7.5 ± 10.0 at 24 months for the SCS and CMM crossover groups, respectively (Fig. 2, Right). For both groups, the point estimate at 24 months is below the value for the general population (13.9). At 24 months, 79.1% (72/91) and 83.3% (40/48) of patients in the SCS and CMM crossover groups reported a 40% reduction from baseline or reported a score of <30 when the patient was clinically catastrophizing (PCS ≥ 30) at baseline. Significant improvements in all 3 PCS subscales were reported at 24 months in the SCS and CMM crossover groups (Supplementary Material Table C2).

**Fig. 3:**
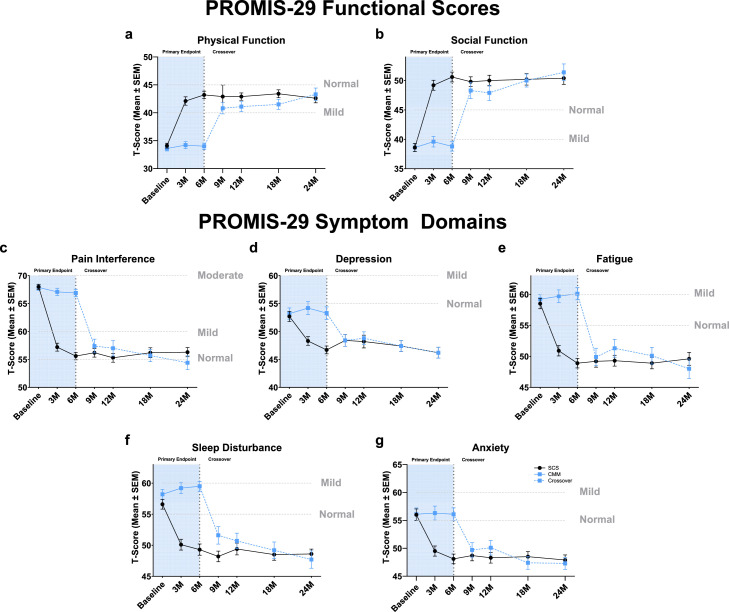
Quality of life/activities of daily living. Improvement in functional ability is denoted by an increase in score. Improvements in symptoms are denoted by a reduction in score. Apart from depression, for which patients reported a mean baseline score within the normal range, baseline symptoms for fatigue, sleep disturbance, and anxiety were mild, and for physical functioning, social roles, and pain interference, were moderate in severity for both groups. At 24 months, all symptomatic domains improved from mild severity to normal levels. Physical functioning improved to mild severity, and social roles and activities improved to normal levels in both groups. Pain interference decreased to mild severity in the SCS group and to normal levels in the CMM crossover groups, although the point estimates at 24 months were not much different in absolute value. PROMIS, patient-reported outcomes measurement information system; SEM, standard error of mean.

**Fig. 4:**
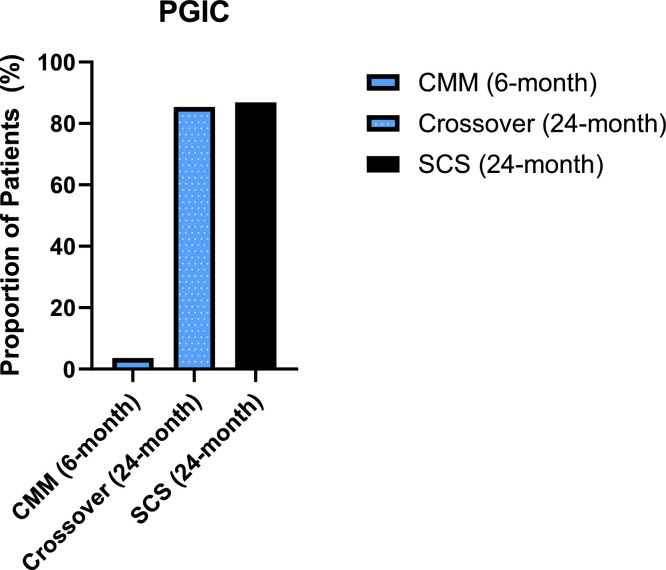
Overall, 86.8% and 85.4% of patients in the SCS and CMM crossover arms reported at least noticeable change in their symptoms with SCS therapy at 24 months. CMM, conventional medical management; PGIC, patient global impression of change; SCS, spinal cord stimulation.

SCS and CMM crossover patients improved in all 7 health domains of the PROMIS-29 questionnaire (Fig. 3). Overall, 89% and 88% of SCS and CMM crossover patients were satisfied with their therapy. On the PGIC, 23.1% (21/91) and 47.3% (43/91) of patients in the SCS group and 33.3% (16/48) and 41.7% (20/48) in the CMM crossover group reported feeling “better” and “a great deal better,” respectively, compared to baseline. Approximately 85% of patients in both groups reported at least a noticeable change from baseline on the PGIC (Fig. 4).

#### Healthcare utilization and medication use

**Fig. 5:**
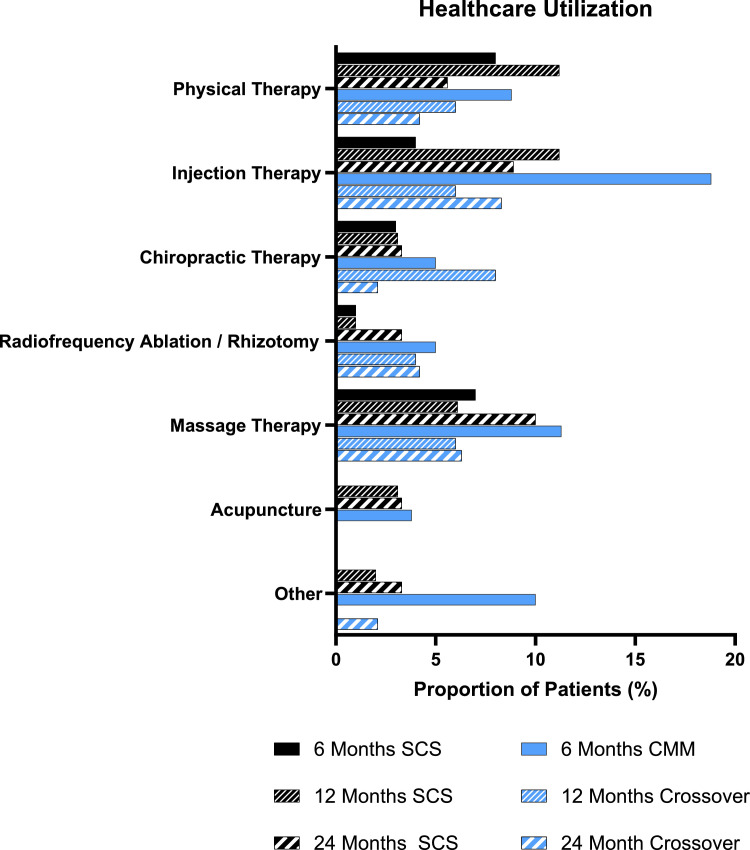
Healthcare utilization at 6, 12, and at 24 months for SCS, CMM, and CMM crossover groups. CMM, conventional medical management; SCS, spinal cord stimulation; PTE, per-treatment evaluable population.

Before participating in the study, most patients in both groups were already using 1 or more pain management therapies. More than 80% of patients reported injection therapy and physical therapy, and 20% to 50% of patients reported acupuncture, massage therapy, radiofrequency ablation, and chiropractic therapy. After 6 months, a smaller proportion of SCS patients used the same therapies or procedures as the CMM group, particularly for injections and other therapies. This trend for SCS patients continued in the CMM crossover group. After 24 months, 5% to 10% of patients were using physical therapy, injection therapy, and massage therapy (Fig. 5).

In both SCS groups, most patients taking opioids were using short-acting opioids (immediate-release formulations). Patients with a morphine equivalent daily dose of more than 50 in the last 30 days prior to enrollment were excluded from participation. About 54% stopped or reduced opioid use, and approximately 40% of the patients stopped or reduced anticonvulsant use. For both medication classes, the percentage of patients who completely discontinued medication increased from 12 to 24 months of follow-up. For the 5 other analgesics—topical medications, antidepressants, antianxiety medications, sleeping aids, and anti-inflammatory medications—most patients remained on their baseline dosage until the final visit (Supplementary Material Table C3). Reduction in healthcare utilization (and related cost-effectiveness of SCS) is detailed in another publication [23]. In short, the average total cost per patient over 6 months was $1,754 in the DISTINCT CMM group and $760 in the SCS group. A comparable real-world CMM cohort had an average cost of $3,502 per patient.

#### Complications

A total of 14 (14/162; 8.6%) and 4 (4/162; 2.5%) patients randomized to the SCS arm reported nonserious adverse device effects (ADEs) and serious adverse device effects (SADEs), respectively, within the first 6 months of the study. Lead migration (4) and IPG site pain (3) were the most common ADEs. Infection (2), numbness (1), and postoperative pain (1) were the reported SADEs. In the final 18 months of the study, 9 patients reported ADEs; no complication occurred in more than 2 patients. After crossover to the end of the study, 2 (2/70; 2.9%) patients reported infection as a SADE. Of 5 ADEs were reported for lead migration (2), infection (2), and seroma (1). The crossover ADE rate was 15.7% (11/70). A total of 8 patients were explanted (out of a total of 170 patients with an implant; 4.7%) due to infection (3), IPG site pain (2), lead damage (1), inadequate pain relief (1), and sleep problems (1). Table 3 summarizes the procedure- and device-related events. By the end of the study, 5 deaths were reported, 3 of which occurred before 12 months of follow-up and 2 after. Independent experts reviewed all deaths and determined that none were related to the device or the procedure.

**Table 3:** SCS procedure- and device-related adverse events.

Event classifier	SCS group			Cross over group		
	No. of events	Patients with events (n/N) %	Event description	No. of events	Patients with events (n/N) %	Event description
	Events through 6 mo					
SADE	2	2/162 (1.2%)	Infection	NA		
	1	1/162 (0.6%)	Numbness			
	1	1/162 (0.6%)	Postsurgical pain			
ADE	5	5/162 (3.1%)	Lead migration			
	3	3/162 (1.9%)	IPG site pain			
	2	2/162 (1.2%)	Infection			
	2	2/162 (1.2%)	Skin reactions			
	1	1/162 (0.6%)	Cerebrospinal fluid leakage			
	1	1/162 (0.6%)	Damaged IPG			
	Events after 6 mo					
SADE	None			2	2/70 (2.9%)	Infection
ADE	2	2/115 (1.7%)	Lead failure	4	4/70 (5.7%)	Lead migration
	2	2/115 (1.7%)	Damaged IPG	3	3/70 (4.3%)	Damaged IPG/lead
	2	2/115 (1.7%)	IPG site pain	2	2/70 (2.9%)	Infection
	1	1/115 (0.9%)	Lead migration	2	2/70 (2.9%)	IPG site pain
	1	1/115 (0.9%)	IPG heating	1	1/70 (1.4%)	Lead failure
	1	1/115 (0.9%)	Loss of stimulation	1	1/70 (1.4%)	Seroma at lead incision site
	1	1/115 (0.9%)	Loss of analgesia			

ADE, adverse device effect; NA, not applicable; IPG, implantable pulse generator; SADE; severe adverse device effect; SCS, spinal cord stimulation.

### Exploratory analysis on the safety and effectiveness of common NS-CLBP diagnoses

The DISTINCT study was not powered to demonstrate the SCS system’s effectiveness for subtypes of NS-CLBP. Furthermore, the number of participants for each subetiology group was not prespecified. Therefore, we limited our analysis to subetiologies that represented at least a quarter of the NS-CLBP patient population: degenerative disc disease, facet joint arthropathy, lumbar radiculopathy, spinal stenosis, and lumbar spondylosis. Chronic nonspecific low back pain was not considered for separate evaluation due to its poorly defined nature, although a significant proportion of patients in each of the identified subetiologies were also diagnosed with a nonspecific pain generator. The NRS, ODI, and PCS results for patients with these subetiologies are presented in Supplementary Material Tables C4 to C6 and Supplementary Material Fig. C1. Overall, improvements on the NRS, ODI, and PCS scales mirrored those of the combined NS-CLBP group for all 5 subetiologies. Patients in the SCS and CMM crossover groups reported nearly identical results at 18 and 24 months on all 3 scales. In none of the 5 subetiologies were more adverse events reported than average.

### Exploratory predictor analysis at 24 months

Only a small number of patients, on average less than 10% of the 5 selected degenerative conditions of NS-CLBP, presented with a single diagnosis. Therefore, exploratory univariable and multivariable regression analyses were performed to investigate whether number of pain diagnoses and/or specific etiologies influenced the chance of success at 24 months. Other clinically important factors considered were the randomization group, baseline NRS, ODI, and PCS scores, presence of unilateral/bilateral leg pain, and PainDetect scores (continuous and categorical). In addition to the clinical outcomes typically assessed in a NS-CLBP population, namely pain and disability, we also included catastrophic thinking in our model, as higher baseline catastrophizing generally predicts poorer long-term pain treatment outcomes [[24], [25], [26]].

After adjusting for covariates in the multivariable analysis, baseline NRS, ODI, and PCS were significant predictors of absolute change on their respective scales, where higher baseline scores were associated with greater improvements. The estimated effect size for baseline NRS scores was 1.06 (95% CI; 0.72, 1.40), or each 1-point increase in baseline NRS corresponds to a 1.06-point improvement in NRS change at 24 months. Similarly, effect sizes for baseline ODI and PCS scores were 0.74 (95% CI; 0.55, 0.93) and 0.94 (95% CI; 0.81, 1.06), respectively. Stepwise regression identified higher baseline PCS (p = .0104) and presence of leg pain (p = .0128) improving the likelihood of super-responder at 24 months, with odds ratio of 2.76 (95% CI; 1.24, 6.15) for leg pain and 1.04 (95% CI; 1.01, 1.07) for PCS (Table 2). In univariable analysis, higher baseline PainDetect and PCS scores were associated with greater absolute change on all 3 outcomes (p < .1) (Table 1).

In univariable analysis, absence of spinal stenosis and fewer pain diagnoses predicted a greater absolute NRS improvement, while lumbar radiculopathy was associated with less improvement in PCS (Table 1). However, in multivariable analysis, neither of the 5 subetiologies, nor fewer pain diagnoses, significantly influenced the odds of greater improvement in NRS, ODI, or PCS, or being classified as super-responder.

### Neuropathic profile of the CLBP condition and efficacy outcomes

The positive predictive effects suggested that the nature of the pain as neuropathic versus nociceptive/mechanical was an important factor for further characterization of the NS-CLBP patient population.

**Fig. 6:**
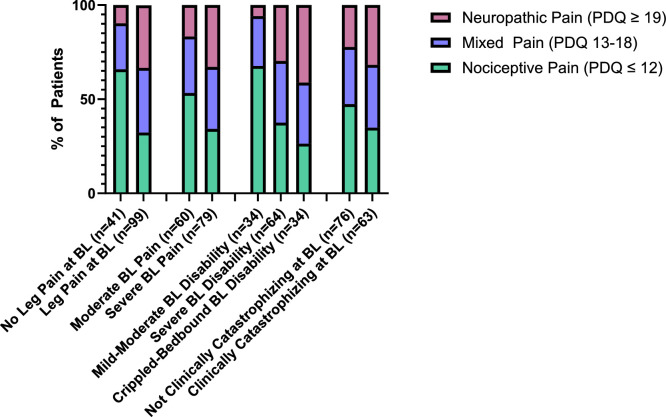
Positive predictive effects according to the PDQ category. Leg pain (Fisher’s exact test; p = .0006) and higher baseline scores for pain (Fisher’s exact test; p = .044) and disability (Fisher’s exact test; p = .0018) were more often associated with a neuropathic pain profile. Moderate pain was defined as baseline NRS scores of 6 or 7 and severe pain was defined as baseline NRS scores of 8 to 10. There was no evidence of an association between pain type and patients who clinically catastrophized at baseline (Fisher’s exact test; p = .2818). BL, baseline; PDQ, PainDetect.

First, we show that leg pain (vs. no leg pain), higher baseline pain (severe vs. moderate pain), and more severe disability (crippled/bedridden vs. severe vs. mild-moderate) were more often associated with patients having a predominantly neuropathic pain profile than with a nociceptive pain profile. Such an association was not found for patients who were clinically catastrophizing (PCS ≥ 30) (Fig. 6).

**Fig. 7:**
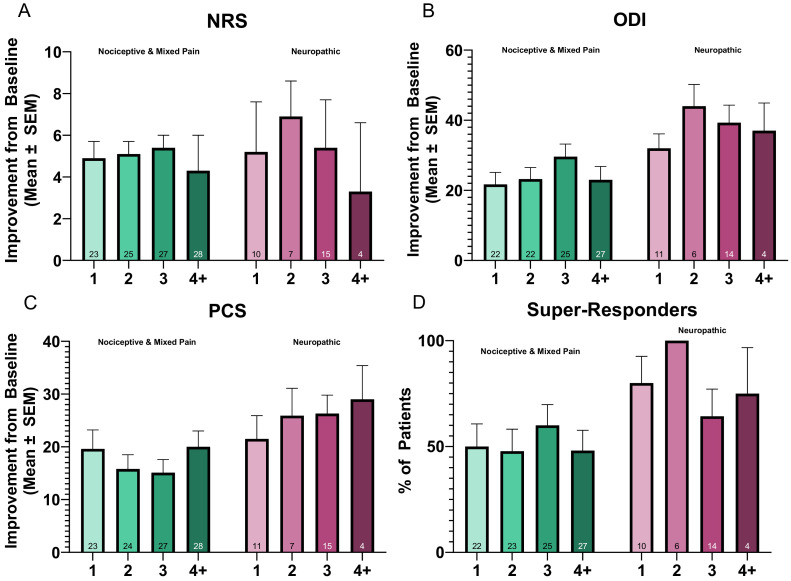
Patients with predominant neuropathic pain (PDQ ≥ 19) reported improvements of 30 to 45 points from baseline on the ODI, compared to 20 to 30 points for patients with nociceptive/mechanical pain or mixed pain (PDQ < 19). Similarly, patients with predominant neuropathic pain reported improvements of 20 to 30 points from baseline on the PCS, compared to 15 to 20 points for patients with nociceptive/mechanical or mixed pain. Overall, 65% to 100% were super-responders when neuropathy was predominant, compared to 50% to 60% for patients with nociceptive/mechanical or mixed pain (B–D). In contrast, patients reported improvements of 5 to 6 NRS points compared to baseline, regardless of the pain type on PDQ (A). NRS, numerical rating scale; ODI, Oswestry Disability Index; PCS, pain catastrophizing scale; SEM, standard error of mean.

Next, we examined the type of pain on the PDQ and the number of diagnoses and their influence on the absolute change in NRS, ODI, and PCS, and on presentation as a super-responder. A logistic regression analysis showed no interaction between the 2 effects. Using a model without interaction, we subsequently found a statistically significant relationship between PDQ category and the change in ODI (p < .001) and PCS (p = .005), and presentation as a super-responder (p = .023). In all 3 analyses, neuropathic pain increased the magnitude of improvement compared with mechanical/nociceptive pain or mixed pain. In contrast, no statistically significant relationship was observed between PDQ category and the change in NRS (p = .4871). Fig. 7 visualizes these results for patients with predominant nociceptive/mechanical or mixed pain type (PDQ < 19) and patients with predominant neuropathic pain (PDQ ≥ 19).

## Discussion

We have previously demonstrated that both groups (SCS and crossover) showed improvements in pain, function, psychometrics, and quality of life after 6 and 12 months of SCS therapy [15,18]. Most patients in the CMM arm chose to trial SCS (crossover) after 6 months, while none of the SCS patients elected to cross over to the CMM arm. Additionally, SCS therapy for NS-CLBP was cost-effective from 2.7 years after implantation, based on lower CMM costs and the additional benefit that SCS brings adjusted for quality-adjusted life years [23,27]. In a subgroup analysis, surgical paddle outcomes demonstrated comparable efficacy and safety outcomes at 12 months as those implanted with percutaneous leads [28]. At a high level, we now demonstrate that this robust outcome is maintained out to 24 months.

This manuscript builds on the available evidence for SCS in the treatment of NS-CLBP and meets the need for longer-term data [29]. Several RCTs have been published to date, demonstrating that SCS is a safe and effective option for patients with NS-CLBP [[30], [31], [32], [33], [34], [35]]. The findings in this manuscript are from the largest patient population with NS-CLBP, encompass a broader range of NS-CLBP etiologies, and are one of only 2 stimulation designs to present data out to 2 years. After 24 and 18 months of burst SCS therapy for the original SCS and crossover groups, NS-CLBP patients showed sustained therapeutic effect across a range of patient reported outcomes related to pain, disability, and the affective/cognitive aspects of pain. Both SCS and crossover groups had virtually identical outcomes at 24 months.

Seventy-seven percent (70/91) and 79% (38/48) of the primary and cross-over group, respectively, reported 50% pain relief at 24 months. The overall mean NRS was sustained at 2.8 and 2.5. Disability improved from a severe-crippling disability to a minimal-moderate disability, with more than double the minimal clinically important difference on this scale in both groups. Functional and symptom domains of the PROMIS-29 profile improved to a mild severity or within normal limits at 24 months. Results on the pain catastrophizing scale decreased significantly below the level expected in the general, nondiseased population. The DISTINCT study is the only published RCT for SCS with 24 months follow up in NS-CLBP patients that collects PCS outcomes to evaluate emotional functioning. Although other RCTs collect EQ-5D outcomes which include a dimension for anxiety/depression, only the index score has been published and, therefore, the impact on psychological distress is not directly available across other studies [[30], [31], [32], [33], [34], [35]]. Sustained improvements compared to the DISTINCT 12-month cohort were also reported for quality of life, healthcare utilization, medication use, and patient satisfaction. At 24 months, nearly 90% were satisfied with the therapy.

The average NS-CLBP patient in the DISTINCT study suffered from chronic pain for 12.5 years and presented with multiple spinal diagnoses. Therefore, we investigated SCS safety and effectiveness for common subetiologies of CLBP to determine whether certain patient populations respond differently to the same intervention. Nonspecific chronic low back pain is characterized by a lack of clearly identifiable features to definitively link a pain-sensing structure to the patient's pain which is 1 prominent reason many of these patients are not candidates for corrective surgery. Approximately 60% of patients in the DISTINCT study were diagnosed with this umbrella term, and literature reports suggest this figure may be as high as 90% to 95% [[36], [37], [38]]. However, due to rigorous patient screening and diagnostic testing, a prerequisite for inclusion in a randomized controlled trial, at least 1 pathoanatomical feature could be identified in more than 85% of participating patients, allowing for a deeper dive into common, specific subetiologies of CLBP. Improvements on the NRS, ODI, and PCS aligned with those of the overall NS-CLBP group, indicating favorable effectiveness for all 5 selected subetiologies: degenerative disc disease, lumbar facet arthropathy, lumbar radiculopathy, lumbar spinal stenosis, and lumbar spondylosis. Similar to the overall results, all SCS and crossover subetiologies reported similar results at 24 months on all 3 scales. Therefore, a precise anatomical diagnosis may not necessarily be a requirement for the effectiveness of SCS in nonsurgical, refractory chronic low back pain. However, the DISTINCT study lacked sufficient statistical power to demonstrate the effectiveness of the SCS system for subtypes of NS-CLBP. We therefore hope that our results can serve as a stimulus for further research into this subject.

Patient selection and treatment optimization is a topic of interest within the SCS field. This is particularly important for patients with NS-CLBP given the uncertainties associated with diagnosis and overlapping sources of discomfort. To this end, exploratory univariate and multivariate regression analyses were performed to identify baseline effects that were associated with success 24 months after implantation. Predictive factors for SCS in the general chronic pain population such as sex, years with pain, tobacco use, and body mass index have been previously published [[39], [40], [41]]. While demographic effects can provide valuable context for understanding group-level trends, they can also lead to biased predictions, reduce the focus on individual-specific factors, and may not result in better-performing models [42,43]. We chose to focus our predictive analysis on actionable clinically relevant effects for NS-CLBP including etiological aspects, diagnosis, and baseline clinical profile.

Regarding the absolute change in NRS, ODI, and PCS, only their respective baseline scores were significant predictors of successful 24-month results. While this analysis is consistent with previous studies of NRS and ODI outcomes, more catastrophizing as a positive predictor contradicts the literature, as higher baseline PCS scores generally predict poorer outcomes [[24], [25], [26],[44], [45], [46]]. While some of this association may be partially explained by regression to the mean, analyses of PCS scores during the randomized phase demonstrated that, although regression to the mean was present, patients receiving SCS had substantially greater improvement than those receiving CMM. Previous posthoc analyses of the Triumph study have shown that burst SCS is as effective in a population with chronic pain and high psychological distress as in chronic pain patients without psychological distress [47]. This information is important in judicious patient selection; specifically, not excluding highly symptomatic and refractory patients from consideration of an SCS trial. Burst stimulation may work by modulating activity in both a suffering component (medial pain pathway) regulated by the dorsal anterior cingulate cortex (dACC) and a pain component regulated by the somatosensory cortex (lateral pain pathway). Studies have confirmed this unique mode of action, particularly through electroencephalogram and positron emission tomography imaging [11,14,48].

Next, we examined predictors for super-responders, ie, patients who were composite responders to NRS, ODI, and PCS. Chronic pain is widely recognized as a multidimensional condition, encompassing sensory, functional, and affective components. Pilitsis et al. [49] showed that individual outcome domains contributed independently to treatment response, supporting the use of a composite framework rather than a single metric [49]. This analysis therefore captures the multidimensional nature of pain and mitigates the limitations of examining predictors for individual responder rates. Patients overall presented with high responder rates and therefore offered less power to detect baseline factors associated with response. Baseline PCS and the presence of leg pain increased the likelihood of a patient being a super-responder, after adjusting for confounders. Each baseline PCS point increase leads to a 4% higher chance of a super-responder and the presence of leg pain increases the chance of a super-responder by almost 3 times at 24 months.

Low back pain can be classified as predominant nociceptive/mechanical, neuropathic, or mixed pain, with a new category of chronic nociceptive pain called nociplastic pain [50]. Leg pain in patients with CLBP although generally felt to be neuropathic, can represent nociceptive referred pain [51]. In neuropathic leg pain, also called sciatica, the pain typically radiates beyond the knee into the foot or toes. This pain may be accompanied by muscle weakness, reflex changes, and tingling in a dermatomal pattern. We demonstrate that leg pain in this population patient was more often associated with neuropathic pain. Neuropathic components in chronic low back pain have been reported to lead to higher pain scores and more severe disability, consistent with our findings [52].

The PainDetect is a questionnaire (PDQ) used to screen for neuropathic pain components in patients with chronic pain, particularly low back pain [53]. PDQ was not used as an inclusion criterion, and as such, we can demonstrate its use as tool for predicting successful long-term outcomes. Patients with likely neuropathic pain reported greater improvements on the PCS and ODI at 24 months and represented a significantly higher percentage of super-responders. Importantly, in the group with predominant nociceptive pain or mixed pain, improvements exceeded the minimal clinically relevant change and were sustained at 24 months. These results were obtained irrespective of number of diagnoses. Higher PDQ scores were also statistically associated with long-term changes on the ODI and VAS after high-frequency SCS treatment in a comparable patient population [54]. Interestingly, these results did not match the pain intensity results at 24 months, where patients reported similar improvements in NRS, regardless of pain type on the PDQ. Our results may call into question the existing dogma about only selecting those with neuropathic pain for SCS therapy. Certainly, these findings merit further study to clarify the role of SCS in this population with nociceptive/mechanical or mixed pain features.

### Limitations of the study

Study limitations that have been presented in previous manuscripts of the DISTINCT study also apply here, including the inability to blind the investigators to the presence of an implantable generator. Although the outcome measures represent the state-of-the-art for evaluating changes in chronic pain patients, cover multiple domains of the pain experience, and correspond to comparable studies to ensure consistency in reporting, they are based on subjective, patient-reported outcomes. These may be biased by an expectation or placebo effect, although literature shows that long-term effects are less susceptible to placebo effects [55,56]. In addition, there was a lack of standardization of noninterventional or interventional therapies that were prescribed during the study. Specifically, for the outcomes at 12, 18, and 24 months, most patients in the CMM arm switching to the SCS arm resulted in the absence of a control at these timepoints. This study design, therefore, functions primarily as an extension of the treated cohort rather than a randomized comparative analysis.

All patients who received an SCS implant first underwent a trial period before proceeding to permanent implantation, which aligns with standard clinical practice and reimbursement requirements for SCS therapy. While the trial requirement may introduce responder enrichment, it also reflects how SCS therapy is delivered in real-world clinical practice. Patients who do not meet minimum responder criteria during trial stimulation are, by design, not eligible for permanent implantation. Accordingly, the long-term outcomes reported in this study are intended to characterize effectiveness among patients who would realistically proceed to implantation in routine care.

The stringent eligibility criteria and the strict, controlled environment of an RCT may not accurately reflect real-world conditions. Moreover, the study required a detailed assessment of the imaging by an independent spinal surgeon to exclude surgically correctable pathology. This was essential to exclude strict surgical indications, thereby strengthening internal validity, while simultaneously acknowledging that less stringent criteria may be applied in practice, which can lead to a less clear delineation. A patient assessment by a spinal surgeon is relevant in practice, as various health insurers require a consultation with a spinal surgeon to determine whether a patient is eligible for a surgical procedure before the patient receives an SCS system. The spinal surgery world continues to refine these criteria as new or different surgical techniques emerge. The current study applies SCS to patients who are not eligible for surgery, whereas patients with persistent postoperative pain have already been extensively studied. Therefore, the evolution of the precise selection criteria for surgery need not change the role of SCS in this general population.

On the other hand, the high-quality data and robust design of the original study provided a reliable basis for the post-hoc analyses. We limited the number of predictor variables to avoid overly complex modeling and clinically irrelevant noise. Alternative approaches may be preferable in future studies designed specifically for prediction modeling, including prespecified or penalized techniques. The presented results were exploratory in nature and are intended to support patient selection and follow-up and the scientific understanding of burst SCS in the treatment of this patient population.

## Conclusions

In this RCT, patients with highly refractory, long-lasting chronic predominately axial low back pain without options for corrective surgery showed a significant and sustained response to passive recharge burst SCS therapy 24 months after implant. The predictor analyses provide a roadmap for patient selection in this patient population. Patients with a severe clinical profile, whether it involves high pain intensity, high mental distress, high levels of disability, the presence of leg pain, and/or a predominantly neuropathic pain profile, will report the greatest improvements. Multiple treatment failures in this patient cohort typically result in worsening pain and increased physical, psychological, and social problems. We demonstrate these characteristics do not constitute an obstacle to a successful outcome with burst SCS therapy.

This challenges long-held beliefs about neuromodulation, including the idea that more severe psychosocial factors lead to poorer long-term patient outcomes. Furthermore, there has long been a presumption that only CLBP patients with a predominantly neuropathic pain profile will benefit from SCS. Although neuropathic pain is an underlying factor increasing the magnitude of success of SCS for NS-CLBP, patients with nociceptive/mechanical pain who had persistent, disabling back pain responded well and sustainably to SCS therapy for up to 24 months. Furthermore, the results for common, specific subetiologies were consistent with those of the entire group of patients with nonsurgical chronic low back pain. This study highlights clinical success in a group suffering disabling back pain for more than a decade, opening the discussion for offering these highly refractory patients SCS therapy and moving SCS therapy earlier in the treatment continuum for refractory, nonsurgical low back pain.

## Data statement

Anonymized individual participant data collected during the DISTINCT study can be shared starting 2 years after publication of the manuscript, with no end date. These data will be available to researchers who provide a methodologically sound proposal for the purposes of achieving specific aims outlined in that proposal. Proposals should be directed to Devyani Nanduri via email devyani.nanduri@abbott.com and will be reviewed by the DISTINCT study management committee. To gain access, data requesters will need to sign a data access agreement and to confirm that data will only be used for the agreed purpose for which access was granted.

## Declaration of competing interests

One or more of the authors declare financial or professional relationships on ICMJE-NASSJ disclosure forms.

## Appendix Supplementary materials

Image, application 1

Image, application 2

Image, application 3

Image, application 4

Image, application 5

Image, application 6

Image, application 7
