# Supplementary figures and images for: Passive recharge burst spinal cord stimulation for the treatment of refractory nonsurgical low back pain: 24-month results from a prospective randomized controlled trial and predictors of success
Source: N Am Spine Soc J. 2026 Jun 8;27:100911. doi: 10.1016/j.xnsj.2026.100911 (PMC13352396; doi:10.1016/j.xnsj.2026.100911)

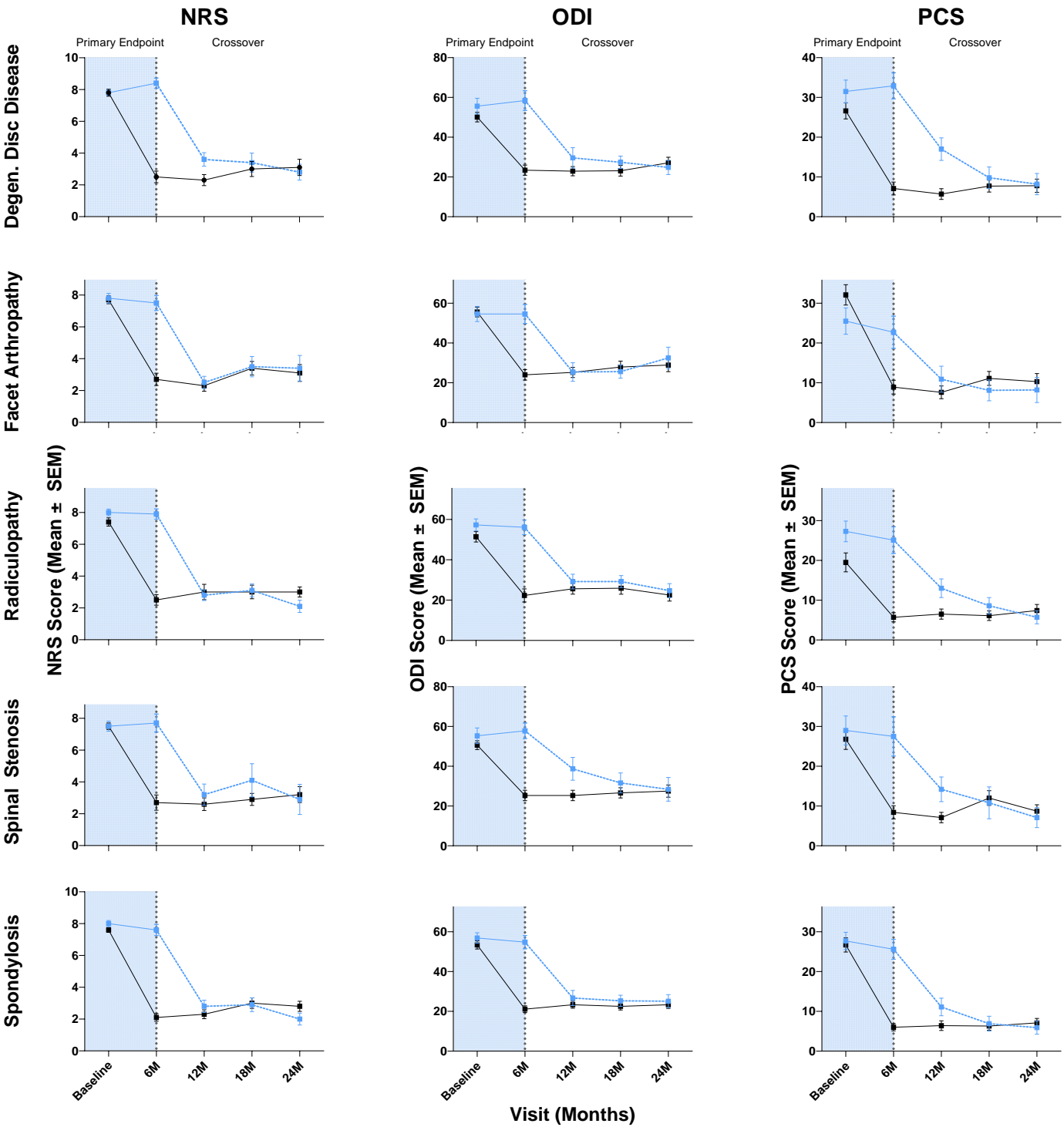

Supplement: Supplementary file 1 [file mmc1.pdf]
