# Supplementary material for: Passive recharge burst spinal cord stimulation for the treatment of refractory nonsurgical low back pain: 24-month results from a prospective randomized controlled trial and predictors of success
Source: N Am Spine Soc J. 2026 Jun 8;27:100911. doi: 10.1016/j.xnsj.2026.100911 (PMC13352396; doi:10.1016/j.xnsj.2026.100911)
Supplement: Supplementary file 2 [file mmc2.docx]

**Supplementary Table C1**. Predictors of response at 24 months; univariable analysis

| **Clinical characteristics^1^** | NRS Change | | ODI Change | | PCS Change | | Super-responder | |
| --- | --- | --- | --- | --- | --- | --- | --- | --- |
|  | Estimate  [95% CI] | p-value | Estimate  [95% CI] | p-value | Estimate  [95% CI] | p-value | Odds Ratio [95% CI] | p-value |
| Randomization Group  (Reference: Crossover) | -0.444  [-1.307, 0.419] | 0.314 | -0.413  [-6.909, 6.084] | 0.901 | 1.009  [-4.185, 6.203] | 0.704 | 0.883  [0.424, 1.838] | 0.739 |
| *Baseline clinical outcomes* | | | | | | | | |
| Baseline NRS score | 1.059  [0.716, 1.402] | <0.0001 | 3.918  [1.111, 6.725] | 0.006 | 0.653  [-1.670, 2.976] | 0.582 | 1.199  [0.864, 1.664] | 0.277 |
| Baseline ODI score | 0.026  [-0.005, 0.056] | 0.096 | 0.742  [0.555, 0.930] | <0.0001 | 0.135  [-0.047, 0.317] | 0.146 | 1.019  [0.993, 1.046] | 0.155 |
| Baseline PCS score | 0.042  [0.009, 0.074] | 0.012 | 0.366  [0.124, 0.608] | 0.003 | 0.938  [0.814, 1.062] | <0.0001 | 1.037  [1.007, 1.068] | 0.0162 |
| *Pain diagnosis* | | | | | | | | |
| Absence of degenerative disc disease | 0.357  [-0.516, 1.230] | 0.423 | 4.089  [-2.407, 10.584] | 0.219 | -1.090  [-6.338, 4.158] | 0.684 | 1.077  [0.517, 2.242] | 0.843 |
| Absence of lumbar facet arthropathy | 0.692  [-0.232, 1.617] | 0.144 | 3.789  [-3.158, 10.736] | 0.286 | -0.814  [-6.403, 4.775] | 0.775 | 1.231  [0.564, 2.685] | 0.602 |
| Lumbar radiculopathy | 0.090  [-0.765, 0.944] | 0.837 | 3.827 [-2.514, 10.168] | 0.238 | -4.965  [-10.047, 0.118] | 0.057 | 1.080  [0.526, 2.214] | 0.834 |
| Absence of Lumbar spinal stenosis | 0.823  [-0.135, 1.781] | 0.094 | 5.567  [-1.636, 12.769] | 0.132 | 0.454  [-5.353, 6.260] | 0.878 | 1.668  [0.742, 3.754] | 0.216 |
| Lumbar spondylosis | 0.272  [-0.556, 1.101] | 0.520 | 4.968  [-1.168, 11.104] | 0.114 | 2.125  [-2.842, 7.092] | 0.402 | 1.505  [0.748, 3.029] | 0.252 |
| Decreasing Number of Pain Diagnoses | 0.252  [-0.028, 0.532] | 0.078 | 0.409  [-1.664, 2.482] | 0.699 | -0.059  [-1.749, 1.632] | 0.946 | 1.122  [0.888, 1.418] | 0.335 |
| Had ≤ 2 Pain Diagnoses | 0.393  [-0.430, 1.216] | 0.350 | -3.226  [-9.373, 2.920] | 0.305 | -0.787  [-5.737, 4.164] | 0.756 | 1.080  [0.538, 2.166] | 0.829 |
| Had ≤ 3 Pain Diagnoses | 1.165  [0.206, 2.124] | 0.018 | 4.062  [-3.170, 11.294] | 0.272 | -1.997  [-7.857, 3.863] | 0.504 | 1.406  [0.626, 3.161] | 0.410 |
| Presence of Leg Pain (Unilateral/Bilateral) | 0.480  [-0.419, 1.380] | 0.296 | 7.914  [1.247, 14.580] | 0.021 | 3.501  [-1.925, 8.928] | 0.207 | 2.500  [1.156, 5.407] | 0.020 |
| *PainDetect questionnaire (reference for categorical scores: Nociceptive/Mechanical Pain (≤ 12))* | | | | | | | | |
| Continuous scores | 0.0524  [-0.005, 0.110] | 0.074 | 0.853  [0.452, 1.254] | <0.0001 | 0.548  [0.213, 0.884] | 0.001 | 1.069  [1.015, 1.126] | 0.011 |
| Categorical scores  Mixed Pain (PDQ 13-18) | 0.504  [-0.456, 1.464] | 0.345 | 3.499  [-3.366, 10.364] | 0.0005 | 1.774  [-3.896, 7.445] | 0.024 | 0.978  [0.437, 2.189] | 0.048 |
| Categorical scores  Neuropathic pain (PDQ ≥ 19) | 0.711  [-0.308, 1.730] |  | 14.556  [7.360, 21.753] |  | 8.246  [2.279, 14.214] |  | 3.026  [1.170, 7.825] |  |

NRS, Numerical Rating Scale; ODI, Oswestry Disability Index; PCS, Pain Catastrophizing Scale; PDQ, PainDetect Questionnaire

^1^For clarity, effects are presented as factors that increased the likelihood of greater absolute change or a higher responder rate for most outcomes. For example, if a sub-etiology decreased the likelihood of success, it is presented as "absence of etiology".
