# Supplementary material for: Passive recharge burst spinal cord stimulation for the treatment of refractory nonsurgical low back pain: 24-month results from a prospective randomized controlled trial and predictors of success
Source: N Am Spine Soc J. 2026 Jun 8;27:100911. doi: 10.1016/j.xnsj.2026.100911 (PMC13352396; doi:10.1016/j.xnsj.2026.100911)
Supplement: Supplementary file 3 [file mmc3.docx]

**Supplementary Table C2.** Outcomes of three subscales of the Pain Catastrophizing Scale (PCS);
Mean ± standard deviation (n)

|  | SCS | | | | | CMM | | CMM-Crossover | | |
| --- | --- | --- | --- | --- | --- | --- | --- | --- | --- | --- |
|  | Baseline | 6M | 12M | 18M | 24M | Baseline | 6M | 12M | 18M | 24M |
| PCS Rumination | 10.3 ± 4.7 (115) | 2.9 ± 3.5 (102) | 3.2 ± 4.1 (97) | 3.0 ± 4.0 (93) | 3.1 ± 4.0 (91) | 10.0 ± 4.6 (101) | 9.7 ± 4.7 (81) | 4.5 ± 4.5 (50) | 3.4 ± 4.1 (49) | 2.9 ± 4.2 (48) |
| PCS Magnification | 5.1 ± 3.7 (115) | 1.5 ± 2.1 (102) | 1.4 ± 2.2 (97) | 1.6 ± 2.2 (93) | 1.5 ± 2.0 (91) | 5.3 ± 3.5 (101) | 5.2 ± 3.6 (81) | 2.0 ± 2.5 (50) | 1.5 ± 1.7 (49) | 1.3 ± 2.0 (48) |
| PCS Helplessness | 12.3 ± 6.5 (115) | 2.9 ± 3.5 (102) | 3.1 ± 4.4 (97) | 3.4 ± 4.5 (93) | 3.3 ± 4.1 (91) | 12.3 ± 5.5 (101) | 11.2 ± 6.5 (81) | 5.2 ± 4.9 (50) | 3.3 ± 4.0 (49) | 3.3 ± 4.5 (48) |

CMM, conventional medical management; PCS, Pain Catastrophizing Scale; SCS, Spinal Cord Stimulation.
