# Supplementary material for: Passive recharge burst spinal cord stimulation for the treatment of refractory nonsurgical low back pain: 24-month results from a prospective randomized controlled trial and predictors of success
Source: N Am Spine Soc J. 2026 Jun 8;27:100911. doi: 10.1016/j.xnsj.2026.100911 (PMC13352396; doi:10.1016/j.xnsj.2026.100911)
Supplement: Supplementary file 4 [file mmc4.docx]

**Supplementary Table C3**. Pain medication usage at follow-up for the SCS groups, CMM (at 6 months), and CMM crossover group (after 6 months)

|  | SCS | | | | CMM | CMM Crossover | | |
| --- | --- | --- | --- | --- | --- | --- | --- | --- |
|  | 6 Months | 12 Months | 18 Months | 24 Months | 6 Months | 12 Months | 18 Months | 24 Months |
| Opioids | | | | | | | | |
| Discontinued | 26.8% (11/41) | 27.9% (12/43) | 28.2% (11/39) | 35.9% (14/39) | 13.8% (4/29) | 15.8% (3/19) | 33.3% (6/18) | 29.4% (5/17) |
| Decreased | 17.1% (7/41) | 25.6% (11/43) | 20.5% (8/39) | 17.9% (7/39) | 6.9%  (2/29) | 26.3% (5/19) | 16.7% (3/18) | 23.5% (4/17) |
| Same | 46.3% (19/41) | 37.2% (16/43) | 35.9% (14/39) | 20.5% (8/39) | 62.1% (18/29) | 36.8% (7/19) | 33.3% (6/18) | 29.4% (5/17) |
| Increased | 7.3%  (3/41) | 4.7%  (2/43) | 12.8% (5/39) | 20.5% (8/39) | 13.8% (4/29) | 10.5% (2/19) | 11.1% (2/18) | 5.9%  (1/17) |
| Started | 2.4%  (1/41) | 4.7%  (2/43) | 2.6%  (1/39) | 5.1%  (2/39) | 3.4%  (1/29) | 10.5% (2/19) | 5.6%  (1/18) | 11.8% (2/17) |
| Anti-convulsants | | | | | | | | |
| Discontinued | 19.0% (8/42) | 28.9% (13/45) | 34.1% (14/41) | 36.6% (15/41) | 20.0% (4/20) | 23.8% (5/21) | 28.6% (6/21) | 33.3% (6/18) |
| Decreased | 9.5%  (4/42) | 4.4%  (2/45) | 4.9%  (2/41) | 2.4%  (1/41) | 0.0%  (0/20) | 0.0%  (0/21) | 0.0%  (0/21) | 5.6%  (1/18) |
| Same | 45.2% (19/42) | 37.8% (17/45) | 34.1% (14/41) | 24.4% (10/41) | 55.0% (11/20) | 38.1% (8/21) | 28.6% (6/21) | 22.2% (4/18) |
| Increased | 21.4% (9/42) | 20.0% (9/45) | 19.5% (8/41) | 19.5% (8/41) | 10.0% (2/20) | 14.3% (3/21) | 14.3% (3/21) | 11.1% (2/18) |
| Started | 4.8%  (2/42) | 8.9%  (4/45) | 7.3%  (3/41) | 17.1% (7/41) | 15.0% (3/20) | 23.8% (5/21) | 28.6% (6/21) | 27.8% (5/18) |
| Topical medication | | | | | | | | |
| Discontinued | 11.5% (3/26) | 14.3% (3/21) | 22.2% (4/18) | 6.3%  (1/16) | 17.9% (5/28) | 33.3%  (3/9) | 11.1%  (1/9) | 30.0% (3/10) |
| Decreased | 0.0%  (0/26) | 4.8%  (1/21) | 0.0%  (0/18) | 6.3%  (1/16) | 7.1%  (2/28) | 0.0%  (0/9) | 0.0%  (0/9) | 0.0%  (0/10) |
| Same | 46.2% (12/26) | 57.1% (12/21) | 61.1% (11/18) | 75.0% (12/16) | 50.0% (14/28) | 55.6%  (5/9) | 66.7%  (6/9) | 40.0% (4/10) |
| Increased | 3.8%  (1/26) | 4.8%  (1/21) | 5.6%  (1/18) | 0.0%  (0/16) | 3.6%  (1/28) | 0.0%  (0/9) | 0.0%  (0/9) | 0.0%  (0/10) |
| Started | 38.5% (10/26) | 19.0% (4/21) | 11.1% (2/18) | 12.5% (2/16) | 21.4% (6/28) | 11.1%  (1/9) | 22.2%  (2/9) | 30.0% (3/10) |
| Anti-depressants | | | | | | | | |
| Discontinued | 4.9%  (2/41) | 9.8%  (4/41) | 2.6%  (1/39) | 5.3%  (2/38) | 0.0%  (0/23) | 0.0%  (0/14) | 0.0%  (0/14) | 33.3% (5/15) |
| Decreased | 2.4%  (1/41) | 2.4%  (1/41) | 2.6%  (1/39) | 0.0%  (0/38) | 0.0%  (0/23) | 0.0%  (0/14) | 7.1%  (1/14) | 0.0%  (0/15) |
| Same | 90.2% (37/41) | 78.0% (32/41) | 84.6% (33/39) | 86.8% (33/38) | 95.7% (22/23) | 85.7% (12/14) | 78.6% (11/14) | 53.3% (8/15) |
| Increased | 0.0%  (0/41) | 2.4%  (1/41) | 0.0%  (0/39) | 5.3%  (2/38) | 4.3%  (1/23) | 7.1%  (1/14) | 7.1%  (1/14) | 6.7%  (1/15) |
| Started | 2.4%  (1/41) | 7.3%  (3/41) | 10.3% (4/39) | 2.6%  (1/38) | 0.0%  (0/23) | 7.1%  (1/14) | 7.1%  (1/14) | 6.7%  (1/15) |
| Anti-anxiety medication | | | | | | | | |
| Discontinued | 9.4%  (3/32) | 14.8% (4/27) | 4.3%  (1/23) | 8.3%  (2/24) | 5.9%  (1/17) | 8.3%  (1/12) | 16.7% (2/12) | 25.0%  (2/8) |
| Decreased | 3.1%  (1/32) | 3.7%  (1/27) | 0.0%  (0/23) | 0.0%  (0/24) | 0.0%  (0/17) | 0.0%  (0/12) | 0.0%  (0/12) | 0.0%  (0/8) |
| Same | 81.3% (26/32) | 77.8% (21/27) | 82.6% (19/23) | 79.2% (19/24) | 94.1% (16/17) | 75.0% (9/12) | 58.3% (7/12) | 75.0%  (6/8) |
| Increased | 3.1%  (1/32) | 3.7%  (1/27) | 0.0%  (0/23) | 8.3%  (2/24) | 0.0%  (0/17) | 0.0%  (0/12) | 8.3%  (1/12) | 0.0%  (0/8) |
| Started | 3.1%  (1/32) | 0.0%  (0/27) | 13.0% (3/23) | 4.2%  (1/24) | 0.0%  (0/17) | 16.7% (2/12) | 16.7% (2/12) | 0.0%  (0/8) |
| Sleeping aids | | | | | | | | |
| Discontinued | 7.9%  (3/38) | 2.9%  (1/34) | 8.6%  (3/35) | 18.2% (6/33) | 8.3%  (2/24) | 0.0%  (0/14) | 11.8% (2/17) | 28.6% (4/14) |
| Decreased | 5.3%  (2/38) | 5.9%  (2/34) | 0.0%  (0/35) | 3.0%  (1/33) | 0.0%  (0/24) | 7.1%  (1/14) | 0.0%  (0/17) | 0.0%  (0/14) |
| Same | 78.9% (30/38) | 82.4% (28/34) | 77.1% (27/35) | 69.7% (23/33) | 75.0% (18/24) | 85.7% (12/14) | 70.6% (12/17) | 64.3% (9/14) |
| Increased | 0.0%  (0/38) | 0.0%  (0/34) | 0.0%  (0/35) | 6.1%  (2/33) | 8.3%  (2/24) | 0.0%  (0/14) | 5.9%  (1/17) | 7.1%  (1/14) |
| Started | 7.9%  (3/38) | 8.8%  (3/34) | 14.3% (5/35) | 3.0%  (1/33) | 8.3%  (2/24) | 7.1%  (1/14) | 11.8% (2/17) | 0.0%  (0/14) |
| Anti-inflammatories | | | | | | | | |
| Discontinued | 17.0% (8/47) | 14.0% (6/43) | 15.8% (6/38) | 9.1%  (3/33) | 7.0%  (3/43) | 19.0% (4/21) | 9.5%  (2/21) | 22.7% (5/22) |
| Decreased | 6.4%  (3/47) | 0.0%  (0/43) | 5.3%  (2/38) | 3.0%  (1/33) | 4.7%  (2/43) | 0.0%  (0/21) | 0.0%  (0/21) | 9.1%  (2/22) |
| Same | 63.8% (30/47) | 72.1% (31/43) | 68.4% (26/38) | 75.8% (25/33) | 72.1% (31/43) | 61.9% (13/21) | 66.7% (14/21) | 54.5% (12/22) |
| Increased | 4.3%  (2/47) | 2.3%  (1/43) | 5.3%  (2/38) | 0.0%  (0/33) | 0.0%  (0/43) | 0.0%  (0/21) | 9.5%  (2/21) | 4.5%  (1/22) |
| Started | 8.5%  (4/47) | 11.6% (5/43) | 5.3%  (2/38) | 12.1% (4/33) | 16.3% (7/43) | 19.0% (4/21) | 14.3% (3/21) | 9.1%  (2/22) |

CMM, conventional medical management; SCS, Spinal Cord Stimulation
