# Supplementary material for: Passive recharge burst spinal cord stimulation for the treatment of refractory nonsurgical low back pain: 24-month results from a prospective randomized controlled trial and predictors of success
Source: N Am Spine Soc J. 2026 Jun 8;27:100911. doi: 10.1016/j.xnsj.2026.100911 (PMC13352396; doi:10.1016/j.xnsj.2026.100911)
Supplement: Supplementary file 5 [file mmc5.docx]

**Supplementary Table C4**. Numerical rating scale outcomes for five common sub-etiologies of non-surgical low back pain; mean ± standard deviation (n)

|  | SCS | | | | | CMM | | CMM-Crossover | | |
| --- | --- | --- | --- | --- | --- | --- | --- | --- | --- | --- |
|  | Baseline | 6M | 12M | 18M | 24M | Baseline | 6M | 12M | 18M | 24M |
| Degenerative Disc Disease | 7.8 ± 1.2 (30) | 2.5 ± 2.0 (30) | 2.3 ± 1.9 (30) | 3.0 ± 2.6 (30) | 3.1 ± 2.8 (30) | 7.8 ± 0.8 (16) | 8.4 ± 1.3 (16) | 3.6 ± 1.6 (14) | 3.4 ± 2.4 (16) | 2.8 ± 2.0 (16) |
| Lumber Facet Arthropathy | 7.7 ± 1.2 (24) | 2.7 ± 1.9 (24) | 2.3 ± 1.7 (24) | 3.4 ± 2.1 (24) | 3.1 ± 2.6 (24) | 7.8 ± 1.1 (13) | 7.5 ± 1.7 (13) | 2.5 ± 1.4 (13) | 3.5 ± 2.3 (13) | 3.4 ± 2.9 (13) |
| Lumbar Radiculopathy | 7.4 ± 1.4 (29) | 2.5 ± 1.8 (29) | 3.0 ± 2.6 (29) | 3.0 ± 2.3 (29) | 3.0 ± 1.7 (29) | 8.0 ± 1.0 (22) | 7.9 ± 1.5 (22) | 2.8 ± 1.5 (20) | 3.1 ± 1.9 (22) | 2.1 ± 1.8 (22) |
| Lumbar Spinal Stenosis | 7.5 ± 1.0 (23) | 2.7 ± 2.3 (23) | 2.6 ± 1.9 (23) | 2.9 ± 1.8 (23) | 3.2 ± 2.4 (23) | 7.5 ± 1.0 (10) | 7.7 ± 1.8 (10) | 3.2 ± 2.1 (10) | 4.1 ± 3.3 (10) | 2.9 ± 3.0 (10) |
| Lumbar Spondylosis | 7.6 ± 1.1 (53) | 2.1 ± 1.7 (52) | 2.3 ± 2.0 (51) | 3.0 ± 2.4 (52) | 2.8 ± 2.3 (52) | 8.0 ± 1.0 (26) | 7.6 ± 1.8 (26) | 2.8 ± 1.9 (24) | 2.9 ± 2.2 (26) | 2.0 ± 1.9 (26) |

CMM, conventional medical management; SCS, Spinal Cord Stimulation.
