# Supplementary material for: Passive recharge burst spinal cord stimulation for the treatment of refractory nonsurgical low back pain: 24-month results from a prospective randomized controlled trial and predictors of success
Source: N Am Spine Soc J. 2026 Jun 8;27:100911. doi: 10.1016/j.xnsj.2026.100911 (PMC13352396; doi:10.1016/j.xnsj.2026.100911)
Supplement: Supplementary file 6 [file mmc6.docx]

**Supplementary Table C5**. Oswestry Disability Index outcomes for five common sub-etiologies of non-surgical low back pain; mean ± standard deviation (n)

|  | SCS | | | | | CMM | | CMM-Crossover | | |
| --- | --- | --- | --- | --- | --- | --- | --- | --- | --- | --- |
|  | Baseline | 6M | 12M | 18M | 24M | Baseline | 6M | 12M | 18M | 24M |
| Degenerative Disc Disease | 50.1 ± 13.2 (30) | 23.4 ± 14.1 (30) | 22.9 ± 12.7 (30) | 23.1 ± 14.7 (30) | 27.1 ± 14.9 (28) | 55.6 ± 15.9 (16) | 58.4 ± 19.8 (16) | 29.6 ± 19.2 (14) | 27.4 ± 12.4 (16) | 24.8 ± 14.4 (16) |
| Lumber Facet Arthropathy | 55.6 ± 12.1 (24) | 31.6 ± 17.4 (24) | 25.2 ± 12.6 (24) | 27.9 ± 14.7 (24) | 28.9 ± 16.3 (24) | 54.6 ± 13.8 (13) | -0.0 ± 13.0 (13) | 25.4 ± 16.2 (12) | 25.6 ± 12.0 (13) | 32.5 ± 17.9 (11) |
| Lumbar Radiculopathy | 51.4 ± 14.0 (29) | 22.3 ± 16.9 (27) | 25.6 ± 13.6 (26) | 25.9 ± 16.0 (29) | 22.5 ± 15.9 (28) | 57.3 ± 14.0 (22) | 56.1 ± 17.1 (22) | 29.2 ± 16.3 (20) | 29.2 ± 13.9 (22) | 24.7 ± 16.0 (21) |
| Lumbar Spinal Stenosis | 50.6 ± 10.6 (23) | 25.3 ± 12.5 (23) | 25.3 ± 12.5 (23) | 26.6 ± 12.3 (23) | 27.5 ± 14.4 (22) | 55.3 ± 12.5 (10) | 57.8 ± 12.4 (10) | 38.7 ± 17.2 (9) | 31.6 ± 15.9 (10) | 28.4 ± 17.9 (9) |
| Lumbar Spondylosis | 53.4 ± 15.0 (53) | 21.1 ± 11.8 (50) | 23.3 ± 12.2 (49) | 22.5 ± 13.1 (51) | 23.3 ± 13.1 (50) | 56.9 ± 13.4 (26) | 54.8 ± 17.1 (26) | 26.7 ± 18.3 (23) | 25.3 ± 14.4 (26) | 25.1 ± 15.9 (23) |

CMM, conventional medical management; SCS, Spinal Cord Stimulation.
