# Supplementary material for: Passive recharge burst spinal cord stimulation for the treatment of refractory nonsurgical low back pain: 24-month results from a prospective randomized controlled trial and predictors of success
Source: N Am Spine Soc J. 2026 Jun 8;27:100911. doi: 10.1016/j.xnsj.2026.100911 (PMC13352396; doi:10.1016/j.xnsj.2026.100911)
Supplement: Supplementary file 7 [file mmc7.docx]

**Supplementary Table C6**. Pain Catastrophizing Scale outcomes for five common sub-etiologies of non-surgical low back pain; mean ± standard deviation (n)

|  | SCS | | | | | CMM | | CMM-Crossover | | |
| --- | --- | --- | --- | --- | --- | --- | --- | --- | --- | --- |
|  | Baseline | 6M | 12M | 18M | 24M | Baseline | 6M | 12M | 18M | 24M |
| Degenerative Disc Disease | 26.6 ± 11.1 (30) | 7.1 ± 8.9 (30) | 5.7 ± 7.4 (30) | 7.7 ± 8.2 (30) | 7.8 ± 9.1 (30) | 31.5 ± 11.4 (16) | 32.9 ± 13.2 (16) | 17.0 ± 10.6 (14) | 9.8 ± 10.8 (16) | 8.2 ± 10.7 (16) |
| Lumber Facet Arthropathy | 32.1 ± 12.5 (24) | 8.9 ± 8.5 (24) | 7.6 ± 7.9 (24) | 11.1 ± 8.6 (24) | 10.3 ± 9.9 (24) | 25.5 ± 11.9 (13) | 22.7 ± 14.6 (13) | 10.9 ± 11.8 (13) | 8.1 ± 9.1 (12) | 8.2 ± 11.4 (13) |
| Lumbar Radiculopathy | 19.5 ± 12.8 (29) | 5.7 ± 6.2 (29) | 6.5 ± 6.9 (29) | 6.1 ± 6.5 (29) | 7.4 ± 8.1 (28) | 27.3 ± 12.2 (22) | 25.1 ± 15.7 (22) | 13.0 ± 10.5 (20) | 8.6 ± 9.4 (21) | 5.7 ± 7.7 (22) |
| Lumbar Spinal Stenosis | 26.8 ± 12.3 (23) | 8.4 ± 7.9 (23) | 7.1 ± 6.3 (23) | 12.0 ± 8.9 (23) | 8.7 ± 7.7 (23) | 29.0 ± 11.5 (10) | 27.5 ± 15.6 (10) | 14.2 ± 9.8 (10) | 10.8 ± 12.0 (9) | 7.1 ± 8.0 (10) |
| Lumbar Spondylosis | 26.7 ± 13.0 (53) | 6.0 ± 6.5 (52) | 6.4 ± 8.7 (51) | 6.3 ± 7.6 (52) | 7.1 ± 8.1 (52) | 27.7 ± 11.1 (26) | 25.6 ± 12.8 (26) | 11.1 ± 10.8 (24) | 6.9 ± 9.4 (25) | 5.9 ± 8.4 (26) |

CMM, conventional medical management; SCS, Spinal Cord Stimulation.
